# COVID19 vaccination choice among Iraqi students at Al-Zahraa University for women

**DOI:** 10.12688/f1000research.55552.1

**Published:** 2021-10-06

**Authors:** Hassan Hadi Al Kazzaz

**Affiliations:** 1 Medical and Health Technology College, Al- Zahra University for Women, Karbala, Karbala, Iraq

**Keywords:** COVID-19, vaccine, preference, Iraqi students, refusal.

## Abstract

**Background: **COVID19 vaccine rejection is a global issue that most developing countries face. A study of COVID-19 vaccine preference among Al-Zahraa University female students will pave the way to resolving the issue of vaccine rejection among students. Students' preferences and refusals of the COVID19 vaccine were evaluated to determine the reasons for their decisions.

**Methods: **This study involved 198 students from Al-Zahraa University for women. An observational cross-sectional study was conducted at Al-Zahraa University in Karbala, Iraq, to find out which Health and Medical Technology students preferred the COVID19 vaccine. Tests based on statistics made use of frequency and rate data.

**Results:** Most students (95%) were over the age of 19. The COVID-19 vaccine was rejected by 138 people (70.4%).  A total of 43 students (28.5%) believed that the COVID19 vaccine may not be completely safe. 49.3% of students were not aware of the differences between the various types of vaccines.  Pfizer was the most preferred by 64 (34.8%), AstraZeneca by 17 (9.2%), and Chinse-Sinovac by only 11 (6%). 20 students (16.4%) believed that with the vaccine they could return to life as it was before the COVID-19 pandemic. Covid-19 vaccine acceptance among Al-Zahara University students may be low in part because of myths, and partly because of the fear of side-effects associated with the vaccine.

**Conclusion:**  Information about COVID-19 vaccines should be transparently communicated to the media by health authorities to help the public make informed decisions.

## Introduction

Scientists are searching for vaccines and medicines to combat the current global COVID-19 pandemic. COVID-19's origins and relationship to bats still needs to be uncovered, but according to current scientific thinking, it's origin was not the result of genetic experimentation, but rather of natural selection. ^1^ This disease has become a major source of concern for medical professionals all over the world due to its rapid spread. It is imperative that all health care providers are up to date with COVID-19 information. ^2^ COVID-19 affects the respiratory system and other internal organs in both animals and humans. ^3^

A thoughtful understanding of vaccine hesitation must be established in the specific historical, political, and socio-cultural context in which vaccination takes place, in addition to an understanding of the factors affecting vaccine acceptance at the individual level. Wider variables effecting vaccination hesitation, such as the function of public health and vaccines, should also be considered. ^4^ In developing nations, parents' failure to bring their children to a vaccination session is frequently linked to vaccination service deficiencies, mirroring the common low-quality immunization pattern in many of those countries. The collective non-adherence form of resistance appears to be mostly driven by religious convictions in poor countries. ^5^ Medical students are among the group of frontline healthcare providers most likely to be exposed to COVID-19 patients. In a recent study on American medical students, concern for serious side effects from the vaccine was independently predictive of lower odds of intent to participate in a COVID-19 vaccine trial (AOR = 0.41, *P* = 0.01). ^6^ Attitudes towards the vaccine do appear to be mostly positive amongst health care worker however, in a recent study on French healthcare workers intention to get vaccinated against COVID-19 reached 75%. ^7^ Many countries have researched to develop the coronavirus SARS-COV-2 vaccine. ^8^ Fear, stress, and worry are normal responses to real threats, especially in times of uncertainty and thus some vaccine hesitancy is to be expected. ^9^

This potential hesitancy and lack of acceptance of COVID vaccines is likely to affect vaccination uptake. A lack of trust in the media amongst college students in the digital era could affect the uptake and acceptance of the COVID-19 vaccine. When the world was faced with a new and terrifying epidemic, television played a crucial role in connecting the government with its citizens. This hesitancy is a serious concern as the vaccine becomes effective only when it is accepted and used by the majority of the global population. ^10^

## Methods

198 first-year students in the Department of Anesthesia at the University of Al-Zahra participated in this cross-sectional questionnaire-based study. A Google Classroom event was attended by 198 students resulting in a response rate of (100%). This research study was analytical in nature. The questionnaire was created by the researcher, and a few questions were adapted from another study ^13^ and incorporated into the questionnaire. The period of research and data collection was between Feb. 2021 and August 2021. Questionnaires were distributed among the students using Google Classroom and were returned by the students after completion via the same method. The responses were retrieved as an excel file from google form a questionnaire and imported into the SPSS version.23 ( IBM SPSS Statistics, RRID:SCR_019096) to be analyzed and frequencies and percentages determined. An alternative open-access software would be R ( R Project for Statistical Computing, RRID:SCR_001905). The multiple-choice questionnaire is related to COVID19 vaccination preference. The questionnaire is comprised of four tables with eight questions. Statistical tests used frequency and rates.

### Ethical consideration

Ethical approval was granted by the independent ethics committee of Al-Zahra University in Karbala-Iraq prior to the research, approval number (HREC 35). The requirement for written consent was waived by the ethics committee. To all those who received a questionnaire, consent messages were sent out, outlining the importance of the study, as well as the researcher's right to privacy. Following a brief explanation of the study's objectives, time commitment, confidentiality, and future benefits to their community to each female participant's consent, they verbally agreed to participate in the study.

## Results

The Al-Zahra University for Women's Anesthesia Department has 198 students in its first year. Table 1 shows the participants' demographic and health-related characteristics. Most students were above 19 years (95%). Out of 170 students, 132 (86.2%) were not married. Most of the students were from Karbala (152, 77.9%). 110 people reported that a close family member had been previously diagnosed with COVID-19 (56.1%), Those with a history of other vaccines rejection were (79%). After being offered the COVID19 vaccine, 188 (70.4%) said that they would decline the vaccine if offered. The type of COVID19 vaccine that students prefer is shown in Table 2. Approximately (49.3%) of students didn't know the difference between types of vaccines. In comparison, 64 (34.8%) preferred the Pfizer vaccine, while 17 (9.2%) preferred the AstraZeneca vaccine, and only 11 (6%) favored the Chinse-Sinovac vaccine. Reasons for wanting to be vaccinated against COVID-19 are shown in Table 3. Among the students, 20 (16.4 %t) reported believing that it would enable them to return to a normal life as a reason for wanting to be vaccinated, and 45 (36.7%) had other reasons to accept the vaccine besides those mentioned in this study. A list of reasons for rejecting COVID-19 vaccination is provided in Table 4. 42.5% of students believed that the COVID19 vaccines may not be completely safe, whilst 33 (21.9%) expressed concern over the vaccine's possible side effects.

**Table 1.** The participants' demographic and health-related characteristics (n: %).

Demographic and health-related characteristics	n	(%)
Age (yr.)		
Below 19 yr.	10	5.1
19 yr.	54	32.7
20 yr.	65()	33.2
Above 20 yr.	57	29.1
Marital status		
Married	22	11.2
Unmarried	170	86.7
Separated	3	1.5
Widow	1	0.5
Residency		
KARBALA	152	77.9
Another governorate	43	22.1
Family member ever diagnosed with COVID-19		
Yes	110	56.1
No	86	43.9
Any previous vaccine (other than COVID19) refusal history		
Yes	154	79%
No	41	21
Do you accept the COVID19 vaccine or refuse it?		
Accept	58	29.6
Refuse	138	70.4

**Table 2.** Which type of available COVID19 vaccine will you choose?

The type of available COVID19 vaccine you prefer	N	%
Pfizer	64	34.8
Oxford-AstraZeneca	17	9.2
Chinse-Sinovac	11	6
Do not know difference	92	50

**Table 3.** Reasons for wanting to be vaccinated against COVID-19.

Reasons for wanting to be vaccinated against COVID-19	N	%
I think vaccination can prevent COVID19	8	6.6
I think vaccination can prevent me from being a spreader	16	13.1
I believe I can return to my life before the COVID-19 pandemic	20	16.4
I think that there is no need to do preventive actions	5	4.1
I have a high risk of getting infected with COVID-19 in my work	14	11.5
I wanted to travel	5	4.1
I think that I should get vaccinated because people around me do so	6	4.9
Other reasons	45	36.9

**Table 4.** Reasons for refusing to acquire the COVID19 vaccine.

Reasons for refusal to get vaccinated against COVID19	N	%
I am concerned about potential side effect	33	21.9
I think COVID19vaccine may not be safe	43	28.5
I do not think that COVID19 is dangerous to my life	17	11.3
I think that COVID19 vaccine is not effective	7	4.6
COVID19 can be avoided without immunization by taking precautions	6	4
I am confident I will not get infected with COVID19	12	7.9
I am against vaccination in general	12	7.9
I am afraid of injection	2	1.3
Religious reasons	1	0.7
Other	18	11.9

## Discussion

Recently, the COVID-19 pandemic has resulted in a rise in mortality and morbidity and affected the lives of people around the world. ^11^ In the AL-Snaafi study, ^12^ COVID19 experience in individuals or their families was 523 (48.7%), which was the same in our study. This could occur because of frequent visits between the two neighboring countries. 154 (79%) of the participants had a history of vaccine rejection. 138 people (70.4%) of our participants said that they would reject the COVID19 vaccine. In a recent study of Egyptian medical students, ^13^ 90.5% perceived the importance of vaccination against COVID-19 while in an Italian study of vaccine hesitancy ^13^ only 51.2% reported that they planned to get the COVID-19 vaccine. This might reflect a difference in the degree of vaccine importance concept ( Table 1). The availability of the COVID-19 vaccine has proven critical in containing the COVID-19 pandemic. Acceptance of the COVID-19 vaccination by the general public will result in the success of this program. ^14^

Al-Zahraa medical students prefer Pfizer vaccine 46 (34.8%), Oxford-AstraZeneca vaccine 17 (9.2%), Chinse-Sinovac vaccine 11 (6%), and 92 (50%) were not aware of the differences between the vaccines. In the aforementioned Italian study ^13^ amongst health workers asked about taking the COVID-19 vaccine, only 20% and 24% preferred to take the Oxford-AstraZeneca or Pfizer vaccine, respectively. Participants in a Saudi Arabian study ^15^ preferred the Pfizer-BioNTech vaccine (20.9%), which was lower than the result in our study. Most Iraqis in this study thought Pfizer's vaccination was the best because it had fewer side effects, and this perception may have been influenced by social media promotion.

In Saied's study ^16^ among Egyptian health workers (46.2%) reported preferring Pfizer-BioNTech and in AL-Snaafi's study ^12^ preferred the Pfizer-BioNTech vaccine which is much higher than Al-Zahraa university students' preference for the same vaccine. The difference might be due to the unfamiliarity of Al-Zahraa university students with the vaccine mechanism and other details in addition to the rumors accompanied with the release of the COVID19 vaccine ( Table 2).

In this study, 20 students (16.4%) wanted to be vaccinated against COVID-19 so that they could resume their normal lives. 45 (36.9%) had other reasons not mentioned. In the Yoda study on a Japanese sample 86.4% of survey respondents thought that vaccination was effective and preventive, and 21.3% thought they would be able to resume their normal lives. ^17^ The disparity between the results of the studies could be attributed to higher living standards and distinctive health promotion activity in Japan as compared to Iraq ( Table 3). Furthermore, in our study on the reasons for refusing to be vaccinated against COVID-19, we discovered that 43(28.5%) of the participants were concerned about potential side effects, while 33 (21.5) believed that the COVID-19 vaccine is not safe. In a survey of the Pakistani general public 28.4% felt the COVID-19 vaccination was developed in a relatively short time, while 19.0% thought that the vaccine was not safe and could kill people. ^18^ This indicates that both participants in Iraq and Pakistan have similar myths about the COVID-19 vaccine.

In Yoda's study about 66.5% were concerned about side effects. Nearly 20% did not trust vaccine efficiency. Comparing with this study, the Japanese study shows more hesitation about side effects than in our Iraqi sample ( Table 4).

## Conclusion

As a result of this research, Al-Zahraa university students' low acceptance of the COVID-19 vaccine has been attributed to myths and fear of side effects. This may be due to the belief that Oxford-AstraZeneca vaccines have more side effects than Pfizer vaccines, and the fact that most of them do not know the difference between COVID19 vaccines at Al-Zahraa University. Vaccine refusal in underdeveloped nations should be addressed with public precautions and emergency measures.

## Recommendation

Information provided to the media about COVID-19 vaccines should be transparent, especially in terms of side effects and safety. Government must also build public trust so that people believe in the government's health advice.

## Consent

It was agreed upon by participants that a message would be sent to the entire recipient list of the questionnaire, mentioning the study's significance as well as their freedom to share their information with the researcher while maintaining their privacy. They were given the option of declining to participate, and they were informed that they might withdraw at any point during the research without giving a reason.

This study was approved by the Human Research Ethics committee at Al-Zahraa university, Approval number (HREC 35).

## Data availability

### Underlying data

Zenodo: COVID19 vaccination choice among Iraqi students at Al-Zahraa University for women. https://doi.org/10.5281/zenodo.5457852. ^19^

This project contains the following underlying data:

• Blank Quiz (Responses).xlsx (Spreadsheet of questionnaire responses).
• COVID19 vaccination choice among Iraqi students at Q.pdf (a blank copy of the questionnaire used in the research).

Data are available under the terms of the Creative Commons Attribution 4.0 International license (CC-BY 4.0).
