# Supplementary material for: COVID19 vaccination choice among Iraqi students at Al-Zahraa University for women
Source: F1000Res. 2022 Jun 28;10:1015. Originally published 2021 Oct 6. [Version 2] doi: 10.12688/f1000research.55552.2 (PMC9280114; doi:10.12688/f1000research.55552.2)
Supplement: Peer review history

# Peer review: Reviewer response for version 2

**DOI:** 10.5256/f1000research.135354.r142552

**Published:** 2022-07-13

**Authors:** Mohammad Yasir Essar

**Affiliations:** 1 Faculty of Dentistry, Kabul University of Medical Sciences, Kabul, Afghanistan

The revision made is convincing and I'm okay with the indexing of the article.

Is the work clearly and accurately presented and does it cite the current literature?

Yes

If applicable, is the statistical analysis and its interpretation appropriate?

Yes

Are all the source data underlying the results available to ensure full reproducibility?

Yes

Is the study design appropriate and is the work technically sound?

Yes

Are the conclusions drawn adequately supported by the results?

Yes

Are sufficient details of methods and analysis provided to allow replication by others?

Yes

Reviewer Expertise:

NA

I confirm that I have read this submission and believe that I have an appropriate level of expertise to confirm that it is of an acceptable scientific standard.

# Peer review: Reviewer response for version 2

**DOI:** 10.5256/f1000research.135354.r142551

**Published:** 2022-07-01

**Authors:** Bijaya Kumar Padhi

**Affiliations:** 1 Department of Community Medicine, School of Public Health, Post Graduate Institute of Medical Education and Research (PGIMER), Chandigarh, Punjab and Haryana, India

The authors addressed most of the comments in the revised manuscript. The manuscript may be accepted as per the journal policy.

Is the work clearly and accurately presented and does it cite the current literature?

Yes

If applicable, is the statistical analysis and its interpretation appropriate?

Yes

Are all the source data underlying the results available to ensure full reproducibility?

No

Is the study design appropriate and is the work technically sound?

Yes

Are the conclusions drawn adequately supported by the results?

Partly

Are sufficient details of methods and analysis provided to allow replication by others?

No

Reviewer Expertise:

Public Health

I confirm that I have read this submission and believe that I have an appropriate level of expertise to confirm that it is of an acceptable scientific standard.

# Peer review: Reviewer response for version 1

**DOI:** 10.5256/f1000research.59138.r125307

**Published:** 2022-04-01

**Authors:** Bijaya Kumar Padhi

**Affiliations:** 1 Department of Community Medicine, School of Public Health, Post Graduate Institute of Medical Education and Research (PGIMER), Chandigarh, Punjab and Haryana, India

The authors used an online-based cross-sectional study among students (n=198) to understand students' preferences and refusals of the COVID19 vaccine in Iraq. Though low sample size is a concern, the study demonstrated a high rate of vaccine refusal. The manuscript is well written, data presentations are clear and adequate. My specific comments are as under:

1. Introduction – How this research fills the gap in the literature? Hypothesis is not mentioned.
2. Method section – How were the study samples obtained? Does the study sample reflect the appropriate and representative population? Inclusion and exclusion criteria not mentioned. Data collection procedure not clearly written.
3. Development of study tools should be described in the method section.
4. Psychometrics of the survey instruments should be provided.
5. Discussion section – more details are needed with regard to implications of the findings for clinical audiences.

Is the work clearly and accurately presented and does it cite the current literature?

Yes

If applicable, is the statistical analysis and its interpretation appropriate?

Yes

Are all the source data underlying the results available to ensure full reproducibility?

No

Is the study design appropriate and is the work technically sound?

Yes

Are the conclusions drawn adequately supported by the results?

Partly

Are sufficient details of methods and analysis provided to allow replication by others?

No

Reviewer Expertise:

Public Health

I confirm that I have read this submission and believe that I have an appropriate level of expertise to confirm that it is of an acceptable scientific standard, however I have significant reservations, as outlined above.

## References

[1] : Factors Influencing COVID-19 Vaccination Intentions Among College Students: A Cross-Sectional Study in India. *Front Public Health* .2021;9: 10.3389/fpubh.2021.735902 735902 10.3389/fpubh.2021.735902 34976911PMC8714761

# Response

**Published:** 2022-04-04

**Authors:** HASSAN al-kazzaz

**Affiliations:** Al-Zahraa university for women, Iraq

Dear Sir,

 Thank you so much for your very fruitful notes.

 In regard to:

 No.1 - The hypothesis was most of Alzahraa university students refuse COVID-19 vaccine. The results in this study approved this hypothesis.

 No. 2- the study include all students in the university which represent the university and also represent the inclusion criteria. The inclusion criteria will be all students outside Al Zahrraa university. 

 Also the study used google class room in collection of the data because of the event of COVID-19 pandemic and the students were taking their lessons via intermate. 

 No.3 - About study tools I had used google classroom and statistics that mention in methodology and will be more than happy if you need any other information of interest.

 No.4- Most of the students refuse to do psychometric test because they think it have some discriminatory against gender. The study respects the privacy of the students.

 No.5 - Rejection of Covid19 vaccine is a global problem. The study pointed out that alzahraa students were on the same floor. Governmental and nongovernmental agencies have to take their roles in convincing their communities to accept COVID-19 vaccination through out media and other steps. this will be done through building trust bridges between the people and health authorities. 

 Its my pleasure and great honor to have you as reviewer to my research and hope my answer will be convenient to you. 

 Please do not hesitate to ask any questions which will add to my knowledge new information.

 Waiting for your reply, wishing you all the best,

 Hassan Al Kazzaz

# Peer review: Reviewer response for version 1

**DOI:** 10.5256/f1000research.59138.r121595

**Published:** 2022-03-22

**Authors:** Mohammad Yasir Essar

**Affiliations:** 1 Faculty of Dentistry, Kabul University of Medical Sciences, Kabul, Afghanistan

The study “COVID19 vaccination choice among Iraqi students at Al-Zahraa University for women” by Hassan Hadi Al Kazzaz has investigated the COVID-19 vaccination choice In Al-Zahraa university. I appreciate the author for conducting this important study. However, I would suggest some comments.

 First, please talk about COVID-19 statistics in Iraq and particularly in city where this university is located.

 Second, talk about what is Vaccine Hesitancy in your introduction. Provide a short description. Use these articles for reference if needed. ^1^ ^2^

 Third, in your discussion, try to compare more studies with your findings. One study I would like you to compare could be Nehmat *et al.* ^3^

 Fourth, it would be great if you could add more to the recommendations. For instance, how your findings could help address the problem. How health policymakers can benefit from this.

Is the work clearly and accurately presented and does it cite the current literature?

Yes

If applicable, is the statistical analysis and its interpretation appropriate?

Yes

Are all the source data underlying the results available to ensure full reproducibility?

Yes

Is the study design appropriate and is the work technically sound?

Yes

Are the conclusions drawn adequately supported by the results?

Yes

Are sufficient details of methods and analysis provided to allow replication by others?

Yes

Reviewer Expertise:

NA

I confirm that I have read this submission and believe that I have an appropriate level of expertise to confirm that it is of an acceptable scientific standard, however I have significant reservations, as outlined above.

## References

[1] : Perceived COVID-19 vaccine effectiveness, acceptance, and drivers of vaccination decision-making among the general adult population: A global survey of 20 countries. *PLoS Negl Trop Dis* .16(1) : 10.1371/journal.pntd.0010103 e0010103 10.1371/journal.pntd.0010103 35089917PMC8797205

[2] : Hesitancy in COVID-19 vaccine uptake and its associated factors among the general adult population: a cross-sectional study in six Southeast Asian countries. *Trop Med Health* .2022;50(1) : 10.1186/s41182-021-00393-1 4 10.1186/s41182-021-00393-1 34983692PMC8727234

[3] : Public Willingness and Hesitancy to Take the COVID-19 Vaccine in Afghanistan. *The American Journal of Tropical Medicine and Hygiene* .2021;105(3) : 10.4269/ajtmh.21-0231 713-717 10.4269/ajtmh.21-0231 PMC859231634237016

# Response

**Published:** 2022-04-04

**Authors:** HASSAN al-kazzaz

**Affiliations:** Al-Zahraa university for women, Iraq

Dear Dr Mohamad,

 Thank you so much for your very fruitful notes. I will work on these notes and let you know.

 Wishing you all the best,

 Hassan Al-Kazzaz
